# Evaluating Characteristics and Quality of Mental Health Apps Available in App Stores for Indian Users: Systematic App Search and Review

**DOI:** 10.2196/79238

**Published:** 2025-09-26

**Authors:** Seema Mehrotra, Ravikesh Tripathi, Pramita Sengupta, Abhishek Karishiddimath, Angelina Francis, Pratiksha Sharma, Paulomi Sudhir, T K Srikanth, Girish Rao, Rajesh Sagar

**Affiliations:** 1 Department of Clinical Psychology National Institute of Mental Health and Neurosciences Bengaluru India; 2 E-Health Research Centre International Institute of Information Technology Bangalore Bengaluru India; 3 Department of Epidemiology, Centre for Public Health National Institute of Mental Health and Neurosciences Bengaluru India; 4 Department of Psychiatry All India Institute of Medical Sciences New Delhi India

**Keywords:** mental health apps, mHealth, review of apps, smartphone apps, MHApps for Indian users, India, mobile phones

## Abstract

**Background:**

The mental health app sector in India is expanding rapidly, driven by increasing smartphone usage, growing internet penetration, the popularity of digital initiatives, and heightened recognition of mental health challenges in public discourse. This growth is also influenced by both supply- and demand-side barriers to seeking professional help and the rise of mental health tech startups. While digital mental health solutions provide scalable ways to address unmet needs, concerns persist regarding app quality, privacy, and safety due to rapid market expansion, regulatory challenges, and limited empirical research. We conducted a comprehensive and systematic review of smartphone-based mental health apps accessible to Indian users through app stores.

**Objective:**

This study aims to describe apps in terms of characteristics such as the nature of their functions, involvement of mental health professionals in development, reference to an empirical basis, and inclusion of nudges to seek professional help, as well as to evaluate app quality.

**Methods:**

This systematic review of mental health apps was conducted using the TECH (Target user, Evaluation focus, Connectedness, and Health domain) approach, along with the PASSR (Protocol for App Store Systematic Reviews) checklist. Fifteen search terms covering mental health conditions and therapies were applied to both Google Play and Apple App Store. Identified apps were screened according to predefined inclusion and exclusion criteria and subsequently downloaded for detailed review. Data were extracted based on prespecified parameters. Additionally, app quality was evaluated using the Mobile Application Rating Scale (MARS).

**Results:**

The initial search identified 5827 apps, of which 350 were reviewed in detail after removing duplicates and applying eligibility criteria. Common search terms such as “depression” and “anxiety” yielded nearly a quarter of relevant apps (128/495, 25.9% to 133/497, 26.8%); 62 (17.7%) of the 350 reviewed apps originated from Asia, and 131 (37.4%) focused on a single mental health condition. Multifunction apps (eg, those combining assessment and intervention) constituted the largest category (230/350, 65.7%). Privacy concerns were notable; for example, 54 (15.4%) apps did not mention a data-sharing policy. Most apps were developed by commercial organizations, and 228 (65.1%) did not report involvement of mental health professionals, while 45 (12.9%) mentioned it only cursorily. Only 38 (10.9%) apps referenced empirical research, and more than half did not indicate an empirical basis for their content. Pointers to seek professional help were present in 139 (39.7%) apps, mostly in the form of disclaimers, whereas nudges or motivational prompts to seek help appeared in slightly less than a quarter. Only 105 (30%) apps attempted to dispel mental health myths. Functionality and aesthetics ratings on the MARS were relatively high, but 50 (14.3%) apps scored 3 or lower on the information subscale.

**Conclusions:**

This study is among the first systematic evaluations of mental health apps accessible to Indian users on Google Play and Apple App Store. The findings provide insights to guide future research, app development, and policy making in the digital mental health space.

**Trial Registration:**

International Platform of Registered Systematic Review and Meta-analysis Protocols (INPLASY) INPLASY2024100035; https://inplasy.com/inplasy-2024-10-0035/

**International Registered Report Identifier (IRRID):**

RR2-10.2196/71071

## Introduction

### Background

Digital mental health solutions offer a scalable approach to addressing the unmet mental health needs of the population. When effectively integrated into community-based services and health care delivery systems, they can facilitate early intervention, reduce the burden on mainstream health care, and promote mental well-being through preventive strategies and sustained support for recovery [1,2].

### Mental Health App Sector in India

The mental health app sector in India is witnessing significant growth. India accounts for over 6% of global mental health tech start-ups, with its app market valued at US $112.75 million in 2023 and projected to grow at an annual rate of 18.2% until 2030 [3,4]. This growth is shaped by multiple factors, including a significant treatment gap due to the limited number of trained professionals, unevenly distributed services, and psychosocial barriers that discourage help-seeking (eg, concerns about social stigma, the perception that mental health struggles are temporary, inadequate support from significant others for professional help-seeking, and gaps in awareness about when to seek professional help) [5-8]. At the same time, the rapid increase in smartphone users, improved internet access, growing familiarity with digital platforms, and greater recognition of mental health challenges in public discourse are driving the expansion of the digital mental health market in India [9-14]. In this context, the appeal of digital mental health tools lies in their ease of access, affordability, and the privacy and anonymity they provide.

### Mental Health Apps: Opportunities and Challenges

Mental health apps can serve a wide range of functions, including enhancing mental health literacy, fostering self-awareness of one’s concerns, reducing barriers to seeking help, and providing basic crisis support. They can offer self-help tools for practice, enable access to online support from professionals or supportive communities, facilitate symptom monitoring and reporting to assist health care providers, and create opportunities for blended interventions that integrate digital self-help with brief therapy sessions [15,16].

While mental health apps present significant opportunities, it is equally crucial to recognize and address key challenges and potential risks to maximize their impact for the public good. Globally, about 10,000 mental health apps are available to the public [17]. The rapid and volatile growth of these apps in virtual stores, combined with insufficient regulatory oversight, makes it extremely difficult for both end users and professionals to identify the most suitable options at any given time [18,19]. Moreover, many well-researched apps remain unavailable to the public, while the majority of readily accessible apps lack direct evidence of effectiveness [20,21]. This underscores the importance of ensuring that all apps include evidence-informed content, which becomes especially vital when users rely on them without professional guidance or oversight [22,23]. Furthermore, privacy and security concerns surrounding mental health apps remain a significant issue, as some handle sensitive data without adequate legal and ethical safeguards, such as clear data-sharing policies, strong encryption, and responsible use of permissions sought [24-26].

### Review of Mental Health Apps in App Stores

Evaluation of mental health apps available to the public in app stores has garnered significant research attention in recent years. These reviews have focused on a wide range of aspects, such as specific mental health conditions (eg, depression, social anxiety [27,28]), app types (eg, artificial intelligence chatbots [29]), age-specific apps (eg, youth-focused apps [30]), and apps available in particular regions [31]. They have also examined issues including privacy and security [25], content quality, user engagement and functionality [31,32], content analysis of consumer reviews [33], and compatibility with evidence-based practices [27,34]. Only a handful of reviews have examined mental health apps from India, addressing themes such as app store descriptions, downloads, pricing, user ratings, and privacy policies [26], as well as apps focused on depression [35], tobacco cessation [36], mental health apps in general [37], and suicide prevention [38]. Most of these studies focused on specific mental health concerns and screened approximately 200-700 apps available to Indian users in app stores, ultimately conducting detailed reviews of about 20-40 apps. To the best of our knowledge, no comprehensive review has yet been conducted on mental health apps available in virtual stores for Indian users.

### Objectives

This study aimed to conduct a comprehensive and systematic review of smartphone-based mental health apps available in app stores accessible to Indian users. The specific objectives were to (1) describe the apps in terms of their characteristics—such as function, type of intervention, target mental health conditions, involvement of mental health professionals in development, reference to an empirical evidence base, and inclusion of content that encourages professional help-seeking (nudges); and (2) evaluate the quality of these apps.

## Methods

### Ethical Considerations

As the study was a systematic review of apps available in virtual stores, an exemption was sought and obtained from the Institute Ethics Committee, National Institute of Mental Health and Neuro Sciences (NIMHANS) (No. NIMHANS/EC/(BEH.SC.DIV.)MEETNG/2024, dated February 6, 2024).

### App Review Approach

A framework called “TECH” (Target user, Evaluation focus, Connectedness, and Health) has been specifically developed to formulate research questions and establish eligibility criteria for systematic reviews of health apps. Traditional frameworks such as PICO (Population, Intervention, Comparison, and Outcome) and SPIDER (Sample, Phenomenon of Interest, Design, Evaluation, Research) are better suited for systematic reviews assessing intervention effectiveness or qualitative studies, respectively [39]. The TECH framework consists of 4 key elements: (1) target user, referring to the specific population of interest; (2) evaluation focus, which may include aspects such as app characteristics, quality, usability, techniques, or components; (3) connectedness, indicating whether the app integrates with other apps or services; and (4) health domain, representing the specific health condition or concern being addressed. This approach helps shape research questions and guide eligibility criteria for app reviews. In this study, the “target user” referred to Indian adults interested in using mobile apps to understand or manage mental health concerns. The “evaluation focus” was broad, encompassing descriptive details about app characteristics, content, and quality. Regarding “connectedness,” the review included both stand-alone apps and those connected to services, but excluded apps that integrated with wearables or other apps. The “health domain” was also broad, covering apps that addressed a range of mental health conditions and concerns. The eligibility criteria outlined later in the study were informed by these elements of the TECH framework.

As this review focused on mental health apps rather than research studies on such apps, the PASSR (Protocol for App Store Systematic Reviews) checklist (see Multimedia Appendix 1) was utilized [21]. PASSR is a combination and adaptation of items from the AMSTAR (A Measurement Tool to Assess Systematic Reviews) and PRISMA (Preferred Reporting Items for Systematic Reviews and Meta-Analyses) checklists, and can be applied to the systematic search of app stores for any category of apps [40,41].

### Eligibility Criteria

The following inclusion criteria were applied to select apps for review:

- Apps available in the Google Play or Apple App Store for Indian adults.
- Store descriptions indicating that the app offers guidance on mental health problems or therapy, information, self-help, or support, and is therefore relevant to the review.
- Apps available in English.
- Fully free apps.
- Free trial version of fully paid apps.
- Free-to-use content within apps that include in-app purchases.

Apps available only in Indian languages, without an English version, were documented but not included in the detailed review. Fully free apps were reviewed in their entirety. Fully paid apps were reviewed based on the features accessible during the free trial period. For apps with in-app purchases, only the freely available content was reviewed as part of this study.

The following exclusion criteria were applied:

- Mental health apps that were fully paid and did not offer a free trial period.
- Apps available solely in non-English languages.
- Apps intended exclusively for use by health professionals (eg, for training or education).
- Apps requiring additional wearable devices or sensors.
- Apps focused solely on wellness enhancement without addressing mental health concerns (eg, improving time management, productivity, or relationships).
- Apps available only to research participants of a given study.

In addition, apps that were no longer available for download at the time of detailed review were excluded.

### Rationale for the Eligibility Criteria

The search criteria were established to effectively address the research objectives while ensuring feasibility for this comprehensive review. The search was conducted in Google Play and Apple App Store, which together account for approximately 99% of the Indian market share [42]. Most mental health apps available to Indian users are in English. Apps in Indian languages that also had an English version were planned to be included in the review. Given that India is a multilingual country [43], apps available exclusively in non-English languages were documented but excluded from detailed review, as their assessment would have required language proficiency. Additionally, the vast and diverse range of apps related to general well-being warrants a separate review. To maintain focus and ensure feasibility, this review was limited to apps addressing 1 or more mental health conditions. Apps requiring additional wearable devices were excluded due to accessibility and affordability concerns. Likewise, apps designed specifically for health professionals were not included, as the review aimed to evaluate apps intended for public use.

### Search Strategy

A keyword-driven search was used to identify apps for review. Fifteen keywords were applied in the virtual stores: mental health, depression, anxiety, PTSD, OCD, addiction, schizophrenia, bipolar, BPAD, CBT, ACT, DBT, cognitive behavior therapy, acceptance and commitment therapy, and dialectical behavior therapy. The selection of these keywords was guided by the purpose of this study—to cover a broad range of prevalent mental health concerns, commonly used psychotherapy interventions, and their acronyms. In addition, a broad scan of recent review studies on mental health apps and an initial scan of the app stores informed the finalization of keywords.

A systematic keyword-based search was conducted between October and November 2024 on both the Google Play and Apple App Store. To avoid algorithmic personalization, each term was searched on freshly reset devices with new Gmail or iOS accounts. Search histories were cleared between each keyword search, and reviewers scrolled through all results to the end of the screen, ensuring they went beyond in-feed advertisements. The full search sessions were screen-recorded for verification and saved on secure drives. All apps identified in each search were manually entered into an Excel (Microsoft Corporation) database using the recordings. Four devices (2 Android and 2 iOS) were used. The review was conducted by 4 primary reviewers (AF, P Sharma, AK, and P Sengupta), with support from expert mentors (SM and RT).

### Initial Screening

Duplicate apps were removed from the Excel sheet. The 4 primary reviewers independently screened the apps for eligibility by examining app store descriptions. If an app was available on both Google Play and Apple App Store for Android and iOS, respectively, only the Google Play version was reviewed, and the app was counted once. This screening task was divided among the 4 members of the review team. Any uncertainty regarding eligibility was resolved through joint discussions. If the reviewers were unable to reach a unanimous decision, consultations were held with the 2 expert mentors (SM and RT) to arrive at a final determination. Apps that met the eligibility criteria were then downloaded for detailed review. During the detailed review, any app deemed ineligible was excluded.

### App Description and Quality Evaluation

Each eligible app was explored in detail, and its characteristics were documented across 21 parameters, including privacy, payment information, type of developer, target mental health condition, and mention of empirical research, among others. Table 1 provides a brief description of these parameters and the approach used for coding them.

App quality was evaluated using the Mobile Application Rating Scale (MARS) [44]. This scale consists of 19 items across 4 domains: engagement, functionality, aesthetics, and information quality. Each item is rated on a 5-point Likert scale: (1) inadequate, (2) poor, (3) acceptable, (4) good, and (5) excellent. The total score and the 4 objective domains demonstrate high internal consistency. An additional 4-item subjective quality scale is available; however, it is not included in the total score and was not used in this review.

**Table 1:** Parameters and coding explanations used to evaluate mental health apps.

No.	Parameter	Coding explanation
1	Privacy policy	Recorded as “accessible to users” if it is easily located and readable, such as being available on the app’s home page, footer, or settings menu. Classified as “clearly explained” if the privacy documentation provides explicit information regarding user responsibilities, app scope, legal compliance, data security, confidentiality, and terms of use in a straightforward and nonambiguous manner. “Mention of data-sharing policy with third parties” is recorded if the policy explicitly states whether and how user data may be shared with external entities, including relevant details about who the third parties are and the purposes for which data are shared. “Mention of data retention duration” is recorded if the policy specifies how long user data will be stored, including time frames or the criteria for deletion or anonymization.
2	Provision for deletion of user account/data	Recorded as “yes” if either the privacy policy or in-app settings specify a mechanism enabling users to delete their account or remove personal information from their device.
3	Payment requirements	Categorized as (1) completely free to use, (2) fully paid, or (3) involving in-app purchases.
4	Nature of developer	Classified according to institutional type: (1) academic institution, (2) commercial organization, (3) government agency, or (4) not-for-profit organization. When information provided is insufficient, it is recorded as (5) insufficient information available.
5	Continent of origin	Designated as North America, South America, Asia, Europe, or Oceania, based on the developer’s location.
6	Release year	Coded as either “released before 2020” or “released after 2020.”
7	Last updated	Indicated as “updated within the past 6 months” or “updated 1 year ago or earlier,” according to the app store information.
8	App downloads	Recorded as reported in app stores (eg, ranges or absolute numbers, as available).
9	App reviews/ratings	Extracted from app store data and coded according to the numerical average user rating displayed on the app store. Ratings were categorized as “5,” “4-4.9,” “3-3.9,” and “2-2.9” for the apps.
10	Target age group	Categorized as children (<12 years), adolescents (13-17 years), adults (18 and above), or as the general population if it is open to all.
11	Language	Coded as (1) English only, or (2) English and 1 or more Indian languages.
12	Technical features	Assessed for the presence of the following: sharing capability (eg, social media integration), app community features, password protection, log-in requirement, reminder functionality, and dependence on internet access.
13	Target mental health condition(s)	Labeled as (1) focuses on a single mental health condition, (2) addresses multiple mental health conditions, or (3) not specified.
14	Type of intervention	Coded as (1) cognitive behavioral therapy, (2) third-wave cognitive behavioral therapy, (3) mindfulness or meditation, (4) behavior change approaches, or (5) other therapeutic approach.
15	Involvement of mental health professionals in the development	Labeled as “clearly specified” when names and qualifications/designations of professionals are provided; as “generally mentioned” when involvement is referenced without sufficient detail.
16	Inclusion of myth-busting content	Recorded as “yes” if content explicitly seeks to clarify or correct common myths or stigmatizing beliefs regarding mental health, disorders, or treatment.
17	Mention of crisis support strategies	Recorded as “yes” if the app provides access to helplines, emergency websites, or outlines basic crisis management strategies.
18	Pointers to seek professional help	Recorded as “yes” if the app or its descriptive content explicitly states it is not a replacement for professional help and encourages users to consult a mental health professional as appropriate.
19	Nudges to seek professional help	Recorded as “yes” if the app delivers prompts or suggestions that encourage users to seek professional support (eg, in response to high scores on screening tools or chatbot interactions).
20	Empirical basis of intervention/assessment	Recorded as “yes” if the app’s intervention or assessment content is explicitly described as empirically supported in the app or store description.
21	Empirical research conducted on the app	Recorded as “yes” if any published empirical studies evaluating the app’s usability, effectiveness, or related outcomes are cited within the app’s content or app store description.

### Review Process

All primary reviewers held at least a master’s degree in psychology and had a minimum of 3 years of research experience. For this review, they underwent structured training in the use of the MARS, a validated tool for evaluating the quality of mobile health apps. The training included viewing the official instructional videos, and the authors of MARS were also contacted to enhance conceptual clarity and ensure a consistent interpretation of the rating criteria.

### Phase 1: Individual Review and Expert Discussion for Calibration

Following MARS training, the 4 primary reviewers independently rated 3 randomly selected apps from the pool of eligible apps, after removing duplicates across search terms and app stores. These independent ratings were then discussed in detail, with discrepancies deliberated and resolved in consultation with expert mentors (clinical psychology faculties SM and RT, each with over a decade of professional experience and prior involvement in app evaluations). This phase facilitated a shared understanding and helped identify specific domains where raters needed to refine and align their judgments.

### Phase 2: Joint Review of Three Apps

In this phase, the 4 reviewers jointly evaluated 3 additional apps, discussing each item and rating in real time. This collaborative exercise reinforced shared understanding and facilitated cross-learning among the reviewers. An internal guide was created to provide a common framework for applying a nuanced approach when rating apps in cases of ambiguity or confusion.

### Phase 3: Independent Review With Joint Discussion

Each of the 4 reviewers was then assigned a new set of 6 apps to review independently. Once rated, these apps were brought back for group discussion, during which all discrepancies were carefully analyzed. These discussions played a crucial role in refining rating standards and were instrumental in identifying specific domains—such as tracking-based apps (eg, mood tracking, sobriety tracking) and assessment/screening test-based apps—where scoring discrepancies arose due to differences in interpretation.

Following this, the remaining pool of apps was divided among the reviewers for independent evaluation, using the calibration established in the earlier rounds. For apps that raised minor uncertainties—due to content ambiguity, unclear functionalities, or cross-domain features—other reviewers were consulted. Apps involving a high degree of uncertainty were recorded in a shared “doubtful apps” sheet, primarily concerning eligibility for inclusion. These apps were flagged for expert-level deliberation in the next phase.

### Phase 4: Expert Review

A total of 7 (2%) apps reviewed in detail were selected by the primary review team for evaluation by a team of 2 experts (SM and RT). These apps were purposively chosen to represent different functions, including assessment, monitoring and tracking, information provision, and interventions.

Comparison of expert ratings with those provided by the primary reviewers revealed discrepancies of up to 2 points on the engagement and information subscales of MARS. These discrepancies occurred only in 2 types of apps: monitoring and tracking, and assessment apps. Following recalibration of the rating norms for these app types, the primary reviewers re-evaluated the apps in these categories.

Additionally, all apps flagged as “doubtful” were discussed with SM and RT during this round, and decisions were made regarding their inclusion in the final pool of reviewed apps.

### Phase 5: Supplementary Analyses

The interrater reliability of the MARS ratings was assessed using 10% of the apps randomly selected from the 350 previously reviewed. Each app in this subset was independently evaluated by all four reviewers, and the intraclass correlation coefficient (ICC) was calculated using Mangold International GmbH - ICC Calculator. In addition, this phase included a further detailed evaluation of paid apps. While the primary analysis focused on free apps or the free content of paid apps, we conducted an additional review of paid content within a subsample of subscription-based apps. Of the 350 apps initially reviewed, 226 involved in-app purchases, and 5 were fully paid apps. At the time of this supplementary review, 20 out of the 226 apps involving in-app purchases and 3 out of the 5 fully paid apps were no longer available in the app stores. Thus, a pool of 206 that offered in-app purchases and 2 that were fully paid was available for review of their paid content. We randomly selected 25% of these 206 in-app purchase apps (n=53) from each of the app categories such as intervention, assessment, multifunctional, etc, ensuring adequate representation across categories. The paid content of these 53 apps, along with the two fully paid apps, yielded a total of 55 apps reviewed between October 25 and November 20, 2025, by the same team members who conducted the primary review. Of the initially selected 55 apps, 10 could not be reviewed due to unavailability on app stores or persistent technical issues; these were replaced with alternative apps drawn from the corresponding categories. The final sample comprised 41 multifunctional apps, 6 intervention-based apps, 5 monitoring and tracking apps, and one app each in the assessment, journaling, and informative categories. The review process strictly followed the same protocol used in the evaluation of free versions.

## Results

### Overview of the Review Process

**Figure 1:**
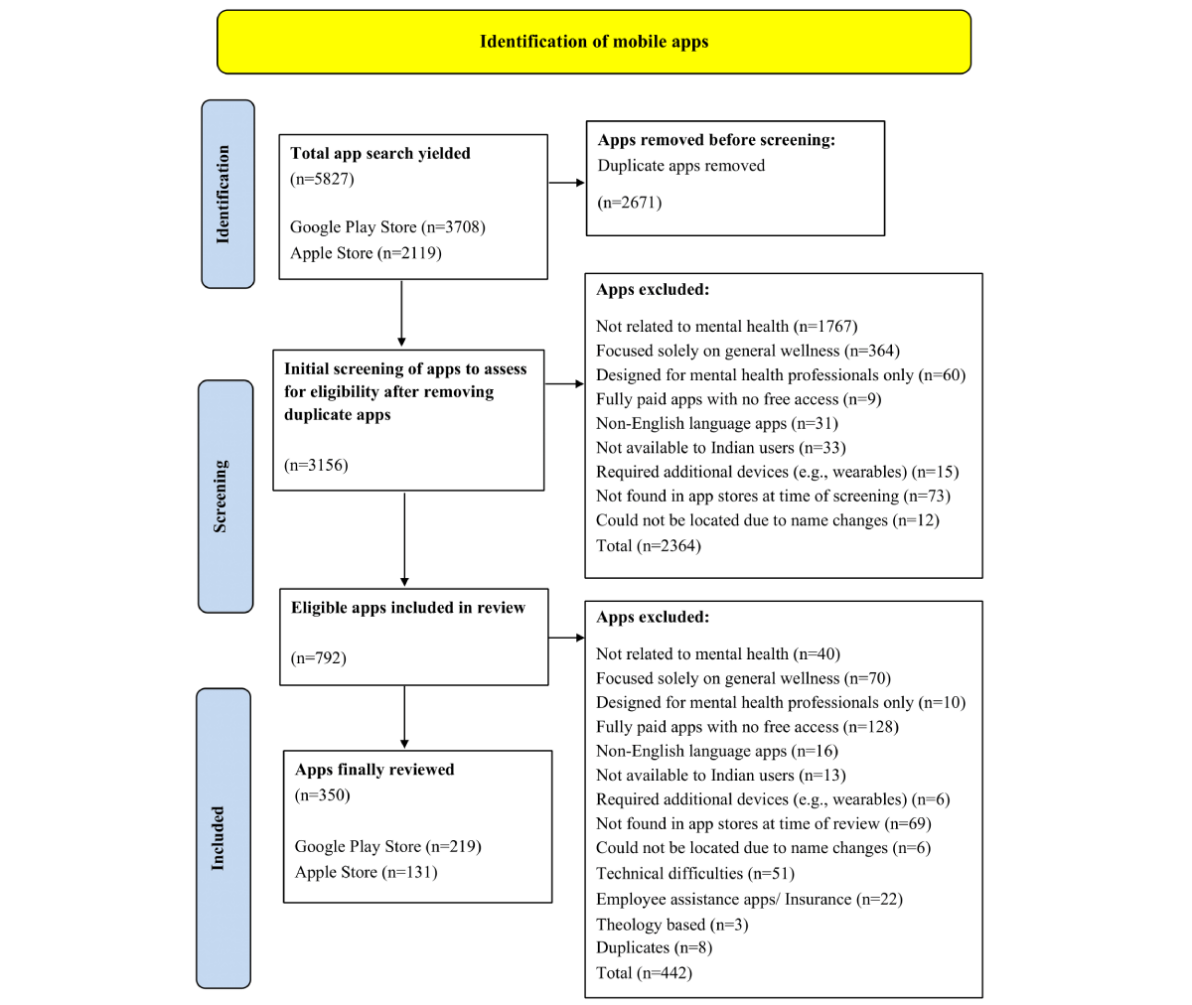
Flowchart of the review process.

The review process is shown in Figure 1.

Table 2 shows that a total of 5827 apps were identified through the keyword-based search. The top 5 keywords yielding the highest number of apps were “mental health” (n=500), “depression” (n=497), “anxiety” (n=495), “OCD” (n=492), and “addiction” (n=484), collectively contributing to more than one-third (n=2468, 42.35%) of the total apps retrieved in the initial search. As indicated in Table 2, for most search terms, only about 20%-30% (for example, the keyword “bipolar” initially yielded 388 apps of which only 78 apps, ie, 20% were retained in the final review, whereas the keyword “cognitive behavioral therapy” initially yielded 303 apps of which 91 apps, ie, 30% were retained in the final review) of the initially retrieved apps were retained after duplicate removal and eligibility screening, highlighting the high proportion of nonrelevant or duplicate content in app store searches.

Of the apps initially identified, a subset of 350 apps met the predefined inclusion criteria and was deemed eligible for final review. Among these, the largest numbers were contributed by apps retrieved using the keywords “depression,” “anxiety,” “mental health,” “CBT,” and “PTSD.” Of the 350 apps reviewed, 164 were available only on Google Play, 130 only on Apple App Store, and 56 were available on both platforms. In other words, 220 (62.9%) of the 350 reviewed apps were available on Google Play, and 186 (53.1%) were available on Apple App Store. As noted earlier, for apps available on both platforms, only the Google Play version was reviewed to maintain consistency in evaluation.

Tables 3 and 4 highlight the characteristics of the apps across various review parameters.

**Table 2:** Number of apps identified using key search terms.

Apps yielded in the initial search on key search terms^a^	Apps eligible for final review, n (%)^b^
Mental health (n=500)	126 (25.2)
Depression (n=497)	133 (26.8)
OCD^c^ (n=492)	59 (12.0)
Addiction (n=484)	50 (10.3)
Schizophrenia (n=265)	67 (25.3)
Anxiety (n=495)	128 (25.9)
PTSD^d^ (n=431)	101 (23.4)
BPAD^e^ (n=234)	11 (4.7)
Bipolar (n=388)	78 (20.1)
Cognitive behavioral therapy (n=303)	91 (30.0)
Dialectical behavioral therapy (n=250)	68 (27.2)
Acceptance commitment therapy (n=257)	58 (22.6)
CBT^f^ (n=456)	122 (26.8)
DBT^g^ (n=324)	40 (12.3)
ACT^h^ (n=451)	38 (8.4)

^a^The initial search yielded a total of 5827 apps.

^b^Apps eligible for final review were 350. An app could be retrieved under multiple search terms. Hence, the total of frequencies corresponding to apps eligible for final review across all search terms exceeds 350.

^c^OCD: obsessive compulsive disorder.

^d^PTSD: posttraumatic stress disorder.

^e^BPAD: bipolar affective disorder.

^f^CBT: cognitive behavioral therapy.

^g^DBT: dialectical behavior therapy.

^h^ACT: acceptance and commitment therapy.

**Table 3:** Characteristics of reviewed apps on basic parameters (N=350).

Review parameters		Values, n (%)
**Privacy policy**		
	Privacy policy accessible to users	310 (88.6)
	Privacy terms clearly explained	301 (86.0)
	Mention of data-sharing policy with third party	296 (84.6)
	Mention of data retention duration	207 (59.1)
**Mention of the provision for deletion of the user account/information from the users’ device**		
	Yes	181 (51.7)
	Not applicable	1 (0.3)
**Payment requirements**		
	Completely free to use	119 (34.0)
	Fully paid	5 (1.4)
	Involves in-app purchases	226 (64.6)
**Nature of the developer**		
	Academic institutions	8 (2.3)
	Commercial organizations	302 (86.3)
	Government agency	13 (3.7)
	Not-for-profit organizations	15 (4.3)
	Insufficient information available	12 (3.4)
**Continent of origin**		
	North America	93 (26.6)
	South America	5 (1.4)
	Asia	62 (17.7)
	Europe	89 (25.4)
	Oceania	5 (1.4)
	Insufficient information available	96 (27.4)
**Release year**		
	Released after 2020	141 (40.3)
	Released before 2020	69 (19.7)
	Information not available^a^	140 (40.0)
**Last updated (at the time of data collection)**		
	Updated 1 year ago or earlier	127 (36.3)
	Updated within the past 6 months	125 (35.7)
	Information not available^a^	98 (28.0)
**App downloads**		
	Up to 10,000	62 (17.7)
	10,000+	37 (10.6)
	50,000+	16 (4.6)
	100,000 to 10 million	88 (25.1)
	10+ million	7 (2.0)
	Information not available^a^	140 (40.0)
**App reviews/ratings**		
	2-2.9	2 (0.6)
	3-3.9	26 (7.4)
	4-4.9	170 (48.6)
	5	17 (4.9)
	Information not available^a^	135 (38.6)
**Technical aspects^b^**		
	Allow sharing (Facebook, Twitter, etc)	101 (28.9)
	Has an app community	68 (19.4)
	Allows password protection	63 (18.0)
	Requires log-in	163 (46.6)
	Sends reminders	199 (56.9)
	Needs web access to function	214 (61.1)

^a^Information not available: due to insufficient information mentioned for the apps in the app stores.

^b^Frequency of technical aspect does not add up to 350, as 1 app can have more than 1 technical aspect.

**Table 4:** Characteristics of reviewed apps on mental health parameters (N=350).

Review parameters			Values, n (%)
**Mental health condition**			
	Single mental health condition focused	131 (37.4)	
	Multiple mental health condition(s)	159 (45.4)	
	Not mentioned	60 (17.1)	
**Type of intervention^a^**			
	Cognitive behavioral therapy	152 (43.4)	
	Third-wave cognitive behavioral therapies	52 (14.9)	
	Mindfulness/meditation	123 (35.1)	
	Behaviour change–focused approaches	49 (14.0)	
	Other therapeutic approaches	27 (7.7)	
Apps attempt to dispel myths			105 (30.0)
Apps mentioning basic crisis support strategies			94 (26.9)
**Mental health professionals’ involvement in the development of the app**			
	Clearly specified	77 (22.0)	
	Generally mentioned	45 (12.9)	
	Not mentioned	228 (65.1)	
Apps providing pointers to seek professional help when needed			139 (39.7)
Apps providing nudges to seek professional help			80 (22.9)
Apps mentioning empirical basis of intervention/assessment			161 (46.0)
Empirical research on the app mentioned within its in-app content or app store description			38 (10.9)

^a^Frequency of intervention type does not add up to 350, as 1 app can have more than 1 intervention type.

### Privacy Policy, Pricing, and Origin of the Apps

A total of 301 (86%) apps had a clearly stated privacy policy. Nearly half of the apps provided users with the option to delete their information if desired. Approximately two-thirds of the apps included in-app purchases, while slightly more than one-third were completely free to use. Of the 350 apps, 36 (10.3%) were developed by academic institutions, government agencies, or not-for-profit organizations. Half of the apps originated from North America and Europe, whereas one-sixth were developed in Asia.

### Recency, Downloads, and Target Population of the Apps

Of the 350 reviewed apps, 141 (40.3%) were released after 2020; 10 (2.9%) were available in an Indian language in addition to English. Download information was not available for more than one-third of the reviewed apps. Among the remaining apps with available download data, approximately one-quarter had between 100,000 and 10 million downloads, while slightly more than one-quarter had fewer than 50,000 downloads. About half of the apps had a user rating of 4 or higher on app stores, although the number of reviews varied. Based on app store descriptions, 2 apps targeted children under 12 years, while 32 apps targeted adolescents (13-17 years). A total of 120 apps specified a target population of 18 years or older. Some apps targeted multiple age groups. However, 299 apps (85.4%) did not mention a specific target age group in their description.

### Type of Interventions

More than half of the apps provided self-help strategies informed by CBT, dialectical behavior therapy (DBT), acceptance and commitment therapy (ACT), and compassion-focused therapy. As DBT, ACT, and compassion-focused therapy are commonly classified as third-wave CBTs, these interventions were grouped accordingly [45,46]. Mindfulness- and meditation-based self-help apps were coded separately, as they did not explicitly indicate that they were informed by CBT principles. Behavior change–focused approaches comprised 49 (14%) of the 350 apps, including 12-step interventions, motivational enhancement therapy, relapse prevention for addiction, exposure and response prevention, other behavioral therapies, and tracking. A wide range of other therapeutic approaches was mentioned in 27 of the reviewed apps, including hypnotherapy, eye movement desensitization and reprocessing, neurofeedback, trauma-informed approaches, Jungian therapy, existential therapy, schema therapy, psychodynamic therapy, humanistic approaches, safety planning, community/peer support, positive psychology, clinical sexology, Gestalt therapy, and somatic therapy. More than two-thirds of the apps did not include content specifically aimed at dispelling myths about mental health. Only about one-fourth of the apps provided crisis support strategies.

### Involvement of Mental Health Professionals and Encouragement for Professional Help-Seeking

Of the 350 reviewed apps, 228 (65.1%) did not indicate whether mental health professionals were involved in their development. Pointers to seek professional help were coded when apps included disclaimers stating that they are neither diagnostic tools nor substitutes for professional assistance. Nudges to seek professional help were coded when an app went beyond disclaimers to actively encourage users to seek professional support based on assessment results or user inputs. More than half of the apps included neither pointers nor nudges to seek professional help.

Table 5 presents the classification of the apps included in the final review. Apps were categorized as “interventions” if they focused on providing therapeutic strategies intended to help users. Apps that focused exclusively on tools for recording and monitoring parameters such as thoughts, moods, or behaviors over time, to gain insights, were classified under “monitoring and tracking.” The “assessment” category included apps that exclusively provide screening tools or tests. Apps that offered only information or resources on mental health or mental health conditions were classified as “informative/educative.” The “other” category comprised artificial intelligence–assisted tools, journaling/diary apps, community support apps, game-based tools, and apps for connecting users to therapists.

Apps were classified as “multifunction” if they combined 2 or more single functions. Among the 230 multifunction apps, the most common functions were monitoring/tracking (186/230, 80.9%), interventions (177/230, 77%), and information/education (130/230, 56.5%). Other functions available in multifunction apps were journaling, assessments, community support, artificial intelligence–based support, and connecting with or finding a therapist.

**Table 5:** Classification of reviewed apps (N=350).

Apps category		Values, n (%)
**Single function**		120 (34.3)
	Interventions (based on therapeutic approaches)	44 (12.6)
	Assessment	24 (6.9)
	Monitoring and tracking	33 (9.4)
	Informative/educative	12 (3.4)
	Other	7 (2.0)
Multifunction		230 (65.7)

Table 6 presents the mean and SD of the final reviewed app categories on MARS subscales and overall average ratings. The overall MARS average rating for all 350 apps was 4.09 (SD 0.44), with a range of 2.52-4.95. Intervention apps received the highest overall MARS average rating, while informative/educative apps scored the lowest. Assessment apps had the lowest mean rating for engagement, whereas multifunction apps scored the highest. All app categories had a mean rating above 4 on the Functionality subscale. The informative/educative category received the lowest average rating on the MARS aesthetics subscale.

**Table 6:** MARSa subscale ratings of reviewed app categories.

App category and MARS subscales		Mean (SD)
**Multifunction (n=230)**		
	Engagement	3.92 (0.58)
	Functionality	4.44 (0.55)
	Aesthetics	4.28 (0.53)
	Information	3.91 (0.49)
	MARS average rating	4.14 (0.43)
**Assessment (n=24)**		
	Engagement	2.89 (0.47)
	Functionality	4.69 (0.49)
	Aesthetics	3.92 (0.51)
	Information	3.60 (0.64)
	MARS average rating	3.77 (0.41)
**Intervention (n=44)**		
	Engagement	3.88 (0.74)
	Functionality	4.72 (0.47)
	Aesthetics	4.30 (0.52)
	Information	3.95 (0.54)
	MARS average rating	4.21 (0.44)
**Informative/educative (n=12)**		
	Engagement	3.10 (0.62)
	Functionality	4.36 (0.67)
	Aesthetics	3.49 (0.62)
	Information	3.65 (0.26)
	MARS average rating	3.65 (0.34)
**Monitoring and tracking (n=33)**		
	Engagement	3.47 (0.48)
	Functionality	4.62 (0.41)
	Aesthetics	4.04 (0.39)
	Information	3.74 (0.37)
	MARS average rating	3.97 (0.28)
**Other (n=7)**		
	Engagement	3.68 (0.72)
	Functionality	4.44 (0.26)
	Aesthetics	4.42 (0.42)
	Information	3.50 (0.39)
	MARS average rating	4.01 (0.33)

^a^MARS: Mobile Application Rating Scale.

The mean ratings on the information subscale of MARS were below 4 for all app categories; 50 (14.3%) reviewed apps received ratings of 3 or lower on the information quality item of the MARS information subscale. Among these, 7 apps received ratings below 3, and their content was examined in detail. Several concerning patterns were observed in these apps regarding the accuracy, safety, and scientific basis of their content. One notable issue was the presence of overstated or misleading claims, portraying the app as a comprehensive therapeutic tool or offering “highly accurate” diagnostic assessments without clear evidence of validation or involvement of qualified mental health professionals. In some instances, there was a notable disconnect between how the app was described (eg, promising depression support or CBT-based interventions) and the features actually provided, which were often limited to basic journaling or habit-tracking functions. Such inconsistencies can be problematic for users seeking targeted mental health support. Additionally, some apps included components that are not widely recognized as interventions without providing users with explanations of their relevance or intended benefits. In a few cases, apps generated alarming feedback (eg, “high suicide risk”) without context or guidance, raising ethical concerns about supporting such users. The absence of crisis support or access to professional help within these apps further compounds the risks. These observations underscore the importance of aligning app content with its stated purpose, ensuring transparency regarding the scientific basis of interventions, and including support mechanisms for users who may be in distress.

Further inspection of MARS ratings revealed that approximately two-thirds of multifunction apps achieved an average rating of 4 or higher, whereas only one-fourth of assessment apps reached this threshold. Three-quarters of intervention apps received ratings of 4 or higher. Less than one-fifth of apps in the informative/educative category achieved ratings of 4 or above, while about half of the monitoring and tracking apps scored 4 or higher on the MARS average rating.

### Results of Supplementary Analyses

ICC analysis demonstrated good to excellent interrater reliability among the four reviewers at the 95% confidence level. Single-measure ICCs ranged from 0.77 (absolute agreement) to 0.80 (consistency), while average-measure ICCs were excellent, ranging from 0.93 to 0.94. These results indicate strong agreement and consistency across raters, particularly when aggregated ratings were used.

A comparison of MARS scores between paid and free versions of the reviewed apps using the Wilcoxon signed rank test indicated no significant difference (z=–1.34; P>.05). The average MARS scores for paid versions ranged from 3.4 to 4.7, while the corresponding free versions ranged from 3.3 to 4.8. These observations suggest that inclusion of paid content in the reviewed apps did not substantially influence the overall MARS ratings. As far as MARS subscales are concerned, the average ratings on functionality and aesthetics were relatively higher (4.3 and 4.6, respectively) compared to ratings on information quality (3.9), mirroring the observations in the primary review.

## Discussion

### Principal Findings

This systematic review provided valuable insights into publicly accessible mental health apps available in virtual stores for Indian users. It identified several key findings related to the emergent app categories, the involvement of mental health professionals in app development, the presence of an evidence base, content quality, the extent to which apps encourage professional help-seeking, and overall app quality as evaluated using the MARS framework. Each of these aspects is discussed in the context of existing literature.

### Reviewed Apps: Emerging Categories

The reviewed apps varied in the functions they offered, their focus on single versus multiple mental health conditions, and the types of interventions provided.

The mental health apps offered a variety of functions, including symptom assessment, information/education provision, symptom or progress monitoring, and interventions. Assessment and monitoring apps were the most common among single-function apps, consistent with previous literature [26,31,35]. Multifunction apps, incorporating 2 or more types of functions (eg, assessment and intervention), constituted the largest category (230/350, 65.7%). This is a positive trend, as such apps may address multiple user needs within a single platform. Further examination of app content revealed that 179 of the 350 (51.1%) apps incorporated multiple intervention strategies, while 126 (36%) were designed around a single component or strategy (eg, breathing exercises). The latter may be useful for individuals with highly specific or well-defined needs, whereas multistrategy apps offer a broader range of options for users to select and utilize according to their needs and relevance.

While 131 (37.4%) apps focused on a single mental health condition (eg, depression), 60 (17.1%) did not specify a focus. The majority targeted multiple mental health conditions, consistent with the overlap observed among common mental health problems in terms of symptoms, comorbidity, and intervention strategies [47-49].

CBT and mindfulness were the most common interventions, followed by DBT, ACT, and motivational enhancement therapy. CBT and related approaches have received considerable attention from researchers and developers in digital mental health, likely due to their widespread popularity and structured framework, which facilitates adaptation into digital self-help tools [50]. The distinction between mindfulness meditation practice and mindfulness-based therapeutic approaches for mental health problems was not always clear, which may be confusing for users. Basic crisis management strategies were included in 94 of the 350 (26.9%) apps, while helpline numbers or emergency service contacts were provided in 109 (31.1%) of the 350 reviewed apps.

Only 105 (30%) of the reviewed apps made a clear effort to dispel common myths about mental health problems. These observations are concerning and are particularly significant in settings where mental health literacy may be relatively low and when apps are accessed by inexperienced or naive users.

### Reflections on, Expert Involvement, Evidence Base, and Content Quality

The majority of the reviewed apps were developed by for-profit organizations. Involvement of mental health professionals in app development was not mentioned in 228 (65.1%) apps, mentioned only cursively in 45 (12.9%), and clearly specified in just 77 (22%) of the 350 apps. Only 38 (10.9%) reviewed apps mentioned empirical research related to the app, with the majority (n=24) focusing on usability, acceptability, and user satisfaction. Direct empirical research on the apps themselves was rare, and more than half of the apps did not provide users with information about the empirical basis for their content. Generating high-quality evidence through rigorous trials is an important goal, but it is a cumulative process that does not always result in the immediate public availability of such apps. Against the backdrop of low mental health literacy [6], the absence of empirically validated or empirically informed content—often due to limited involvement of mental health professionals in app development [1]—further increases challenges and risks for users, who may struggle to assess the validity of app content [25,33].

The validity and quality of app content have been identified as concerns in several studies. For example, one study on depression apps found that only 40% involved a mental health expert in their development [26], while another study on social anxiety apps reported that the content often lacked sufficient information to determine whether it was based on expert validation [28]. Another review of depression apps, examining adherence to evidence-based guidelines, found that 21% of the reviewed apps suggested scientifically unproven treatments [34]. Similarly, a review of suicide prevention apps reported that only one-third included supplementary information on scientifically validated content for users [38]. Another study noted that the number of evidence-based components in apps was not significantly associated with average consumer ratings [32]. Consistent with these findings, existing reviews also indicate the presence of unscientific or misleading information in mental health apps, although this issue has not been comprehensively or thoroughly examined. As the majority of mental health apps available to consumers have not been validated through empirical studies, it is especially important that their content is evidence-informed and developed in collaboration with mental health experts [22].

Despite the increasing number of studies on mobile apps for mental health, previous reviews have highlighted a lack of empirical research on apps publicly available in virtual stores [1,34]. This suggests a gap between research and the accessibility of research-based apps on one hand, and the rapid proliferation of apps with minimal involvement of mental health professionals on the other. The current regulatory landscape for digital mental health needs further development to safeguard the interests of end users [18]. Additionally, there are relatively few implementation studies on mental health apps that could inform real-world adoption, engagement, and effectiveness across different settings [1]. The barriers limiting the public availability of evidence-based or evidence-informed mental health apps must be identified and addressed.

### Encouragement for Professional Help-Seeking

A notable concern was the lack of adequate information, guidance, and prompts to help users of self-help apps recognize when professional support is necessary. This issue is particularly critical in unguided self-help apps, as users may not be sufficiently aware that the app may not be suitable for everyone or that certain situations require transitioning from self-help to professional care. Pointers to seek professional help were present in 139 of the 350 (39.7%) reviewed apps, mostly in the form of disclaimers stating that the apps were not diagnostic tools or substitutes for consultation with a health professional. Nudges or motivational prompts to seek professional help, based on assessment results or user inputs, were noted in slightly less than a quarter of the apps. Given the mental barriers to help-seeking—such as normalization of distress, stigma, and preference for self-reliance—alongside low mental health literacy, particularly in the Indian context [6-8], it is ethically crucial for mental health apps to clearly delineate their scope, including for whom they may be most useful. Beyond providing information or guidance, these apps need to actively nudge users to seek professional help when appropriate, rather than relying solely on self-help [34,35].

### Evaluation of App Quality Using MARS

Existing reviews indicate that most apps score high on functionality (eg, performance, ease of use) and that MARS ratings for functionality and aesthetics tend to correlate significantly with consumer ratings of the respective apps [32,51]. The MARS average rating of all apps reviewed in this study shows a similar trend to that observed in previous studies [51-53].

In this study, all app categories—except informative/educative and assessment apps—had average ratings above 4 on both the functionality and aesthetics subscales. Engagement ratings were highest (close to 4) for multifunction apps and lowest for assessment and informative/educative apps. Average ratings across all app categories were below 4 on the information subscale. A closer examination of apps with low information quality (ratings <3) highlighted several areas of concern, as discussed earlier.

Examining the apps with relatively higher MARS ratings, it was noted that two-thirds or more of the apps in the multifunction and intervention categories had average MARS ratings of 4 or higher. This review considered both MARS ratings and app descriptions based on several other parameters deemed relevant for mental health apps. While the MARS framework provided a consistent and standardized approach to evaluation, certain nuances important for mental health apps were not fully captured within its subscales. Ratings for each subscale were based on individual item scores, which showed some variability. Moreover, the average rating across subscales—where all subscales were weighted equally—tended to be relatively high, particularly because many apps received high scores on functionality and aesthetics. This issue has been noted in previous studies using MARS [54]. These findings are consistent with existing studies on mental health apps assessed using MARS [51,52]. Future studies could benefit from considering additional parameters specific to mental health to achieve a more comprehensive understanding of apps available in virtual stores.

### Additional Observations

A slightly larger proportion of reviewed apps (220/350, 62.9%) were available on Google Play, which may partly reflect the market share of Android phones [42] and the relative ease of hosting mental health apps on this platform, making it appealing to developers [55]. It should be noted that both factors are rapidly evolving, with a growing market for Apple phones in India [56] and changes in the requirements and validation steps for hosting health apps on the Google Play [57]. Nearly 40% of the reviewed apps were released after 2020 (141/350, 40.3), potentially reflecting the rapid advancement of digital health and increased awareness of mental well-being, particularly during and after the COVID-19 pandemic. Common search terms such as “depression” and “anxiety” yielded nearly a quarter of relevant apps (128/495, 25.9% to 133/497, 26.8%) after duplicates were removed and ineligible apps filtered out according to the review criteria. Previous studies have similarly documented low rates of finding relevant apps for specific purposes using keyword-driven searches [28,58,59]. These figures also offer a rough indication of the challenges users may face when trying to find relevant apps using specific keywords.

Of the 350 reviewed apps, 40 (11.4%) did not have a privacy policy accessible to users, and in another 49 (14%), the policy was not clearly explained. Details on data sharing and the option to delete accounts were missing in 54 (15.4%) and 168 (48%) apps, respectively, while the duration of data retention was not specified in 143 (40.9%) apps. These observations raise concerns regarding privacy and security, consistent with findings from other reviews [35,60]. In an Indian review of 50 depression apps, more than one-fourth did not have a privacy policy accessible through the store description, and some apps requested potentially risky permissions (eg, modify or delete USB storage) [26]. Another review of depression apps found that although most included a privacy policy, only one-third presented it to users before account creation [27]. End user reviews have also highlighted privacy concerns [33].

Our review found that several apps required in-app purchases, although evaluating their costs was beyond the scope of this analysis. Notably, only 119 of the 350 (34%) apps were entirely free, which could limit accessibility for users in low- and middle-income countries. Most apps originated from North America and Europe, while in about a quarter of cases, this information was unclear. Of these 350, 62 (17.7%) apps were developed in Asia, and 10 apps were available in an Indian language alongside English. This underscores the need for investment in the development and adaptation of evidence-informed and evidence-based apps that provide socioculturally appropriate content for Indian users.

### Strengths and Limitations

This is one of the first systematic reviews to comprehensively examine mental health apps accessible on Google Play and Apple App Store for Indian users. The review had several strengths, including the use of a broad range of search terms related to mental health conditions and therapies, which ensured comprehensive coverage. Additionally, it incorporated recommended guidelines for systematic app reviews, employed a detailed set of evaluation parameters, and used a standardized rating tool, all of which enhance its potential utility for future research.

Nevertheless, the review had some limitations. First, we were able to evaluate only free apps, apps with free trials, and the freely accessible portions of paid apps. This may have provided an incomplete basis for rating some apps. Second, the review was intentionally limited to apps addressing mental health concerns and therapies, and did not include search terms related to general well-being. This was intentionally done because wellness apps constitute a large and heterogeneous category. However, this approach may have excluded some apps that provide useful information for managing mental health concerns but do not explicitly highlight this in their names or app store descriptions. Third, the detailed and comprehensive nature of the review, combined with the constantly evolving content of apps on virtual stores, imposed constraints on the review strategy. Apps were assigned to 4 different members of the review team for evaluation, rather than being reviewed concurrently by multiple raters. To enhance consistency across evaluators, several measures were implemented: joint training sessions for reviewers; discussions following independent ratings of a subset of apps to establish consensus; joint ratings and deliberations on selected apps; adherence to an internal guideline for MARS ratings; and a cross-validation exercise involving senior faculty members on a selected subsample of apps.

### Future Directions and Conclusions

Overall, the findings of this systematic review of mental health apps available in virtual stores for Indian users provide valuable insights to guide future research, development, and policy making in the digital mental health sector. The limited empirical evidence supporting publicly available apps, coupled with indications of insufficient involvement of mental health professionals in their development, underscores critical gaps that require attention. While further studies are needed to strengthen the evidence base on app effectiveness and related factors, future research should also aim to identify barriers that limit the meaningful involvement of mental health experts in app design and development. A related concern, noted previously, is that many well-researched apps remain unavailable in app stores [19,50]. Efforts to identify and address bottlenecks in deploying rigorously researched apps on virtual platforms, and to promote their adoption across diverse settings, could enhance both the accessibility and impact of mental health apps.

This review highlights a marked underrepresentation of mobile mental health apps from the Asian continent, with just 62 (17.7%) developed in the region and only a handful originating from India. This gap underscores the urgent need for research that evaluates the sociocultural relevance of app content and rigorously tests the effectiveness of publicly available tools within Indian contexts. Addressing this challenge will require concerted efforts, including adequate funding support from local government agencies and other stakeholders, as well as active involvement of mental health professionals in both clinical and research domains. Such engagement is crucial for the development of indigenous apps that incorporate evidence-informed content tailored to local needs.

As the mental health app sector in India continues to expand rapidly, it is increasingly important to strengthen regulatory guidelines that protect user privacy, address safety concerns, and uphold quality standards—measures that are essential for fostering trust in digital mental health solutions. Additionally, exploring public-private partnership models could support the deployment and long-term sustainability of these apps. Equally important are initiatives to improve digital mental health literacy among potential users. With an increasing number of mental health apps of varying quality available in app stores, educating individuals about the scope and limitations of these tools—and guiding them in identifying apps that best meet their needs—can empower users, particularly those unfamiliar with mental health apps, to make informed decisions and maximize the benefits of digital mental health interventions.

## Appendix

Multimedia Appendix 1 PASSR checklist.

## Abbreviations

ACT: acceptance and commitment therapy
AMSTAR: A Measurement Tool to Assess Systematic Reviews
CBT: cognitive behavioral therapy
DBT: dialectical behavior therapy
ICMR: Indian Council of Medical Research
INPLASY: International Platform of Registered Systematic Review and Meta-analysis Protocols
MARS: Mobile Application Rating Scale
PASSR: Protocol for App Store Systematic Reviews
PICO: Population, Intervention, Comparison, and Outcome
PRISMA: Preferred Reporting Items for Systematic Reviews and Meta-Analyses
PTSD: posttraumatic stress disorder
SPIDER: Sample, Phenomenon of Interest, Design, Evaluation, Research
TECH: Target user, Evaluation focus, Connectedness, and Health

## Data Availability

The data of this study will be made available upon request to the corresponding author (SM).
