# Addendum: Evaluating Characteristics and Quality of Mental Health Apps Available in App Stores for Indian Users: Systematic App Search and Review

**DOI:** 10.2196/91934

**Published:** 2026-03-30

**Authors:** Seema Mehrotra, Ravikesh Tripathi, Pramita Sengupta, Abhishek Karishiddimath, Angelina Francis, Pratiksha Sharma, Paulomi Sudhir, T K Srikanth, Girish Rao, Rajesh Sagar

**Affiliations:** 1 Department of Clinical Psychology National Institute of Mental Health and Neurosciences Bengaluru India; 2 E-Health Research Centre International Institute of Information Technology Bangalore Bengaluru India; 3 Department of Epidemiology Centre for Public Health National Institute of Mental Health and Neurosciences Bengaluru India; 4 Department of Psychiatry All India Institute of Medical Sciences New Delhi India

In “Evaluating Characteristics and Quality of Mental Health Apps Available in App Stores for Indian Users: Systematic App Search and Review” [1], the authors made two additions.

First, toward the end of the Methods section, the authors added the following:

***Phase 5: Supplementary Analyses***

The interrater reliability of the MARS ratings was assessed using 10% of the apps randomly selected from the 350 previously reviewed. Each app in this subset was independently evaluated by all four reviewers, and the intraclass correlation coefficient (ICC) was calculated using Mangold International GmbH - ICC Calculator. In addition, this phase included a further detailed evaluation of paid apps. While the primary analysis focused on free apps or the free content of paid apps, we conducted an additional review of paid content within a subsample of subscription-based apps. Of the 350 apps initially reviewed, 226 involved in-app purchases, and 5 were fully paid apps. At the time of this supplementary review, 20 out of the 226 apps involving in-app purchases and 3 out of the 5 fully paid apps were no longer available in the app stores. Thus, a pool of 206 that offered in-app purchases and 2 that were fully paid was available for review of their paid content. We randomly selected 25% of these 206 in-app purchase apps (n=53) from each of the app categories such as intervention, assessment, multifunctional, etc, ensuring adequate representation across categories. The paid content of these 53 apps, along with the two fully paid apps, yielded a total of 55 apps reviewed between October 25 and November 20, 2025, by the same team members who conducted the primary review. Of the initially selected 55 apps, 10 could not be reviewed due to unavailability on app stores or persistent technical issues; these were replaced with alternative apps drawn from the corresponding categories. The final sample comprised 41 multifunctional apps, 6 intervention-based apps, 5 monitoring and tracking apps, and one app each in the assessment, journaling, and informative categories. The review process strictly followed the same protocol used in the evaluation of free versions.

Second, toward the end of the Results section, the following was added:

***Results of Supplementary Analyses***

ICC analysis demonstrated good to excellent interrater reliability among the four reviewers at the 95% confidence level. Single-measure ICCs ranged from 0.77 (absolute agreement) to 0.80 (consistency), while average-measure ICCs were excellent, ranging from 0.93 to 0.94. These results indicate strong agreement and consistency across raters, particularly when aggregated ratings were used.

A comparison of MARS scores between paid and free versions of the reviewed apps using the Wilcoxon signed rank test indicated no significant difference (z=–1.34; P>.05). The average MARS scores for paid versions ranged from 3.4 to 4.7, while the corresponding free versions ranged from 3.3 to 4.8. These observations suggest that inclusion of paid content in the reviewed apps did not substantially influence the overall MARS ratings. As far as MARS subscales are concerned, the average ratings on functionality and aesthetics were relatively higher (4.3 and 4.6, respectively) compared to ratings on information quality (3.9), mirroring the observations in the primary review.

The correction will appear in the online version of the paper on the JMIR Publications website, together with the publication of this correction notice. Because this was made after submission to PubMed, PubMed Central, and other full-text repositories, the corrected article has also been resubmitted to those repositories.
