# Supplementary material for: Evaluating Characteristics and Quality of Mental Health Apps Available in App Stores for Indian Users: Systematic App Search and Review
Source: JMIR Mhealth Uhealth. 2025 Sep 26;13:e79238. doi: 10.2196/79238 (PMC12514412; doi:10.2196/79238)
Supplement: Multimedia Appendix 1 [file mhealth_v13i1e79238_app1.pdf]

Supplementary file 1

Checklist

Protocol for app store systematic reviews (PASSR) Marshall, Dunstan and Bartik (2020)

**Sections and topic**	**Addressed in review**
**Section 1: Conducting an App Store Systematic Review (adapted from PRISMA)**	
**TITLE**	
1. Identify the report as an app store systematic review (and meta-analysis if applicable) in the title.	Yes
**ABSTRACT**	
2. Provide a structured Abstract confirming that the review utilized the PASSR framework, and, as applicable: background, objectives, app store sources, study eligibility criteria, app appraisal and synthesis methods, results, limitations, conclusions and implications of key findings, and systematic review registration number.	Yes
**INTRODUCTION**	
3. In the Introduction, describe the rationale for the app store review in the context of what is already known.	Yes
4. Provide an explicit statement of questions being addressed with reference to an adapted PICOS model for app store reviews (all of these may not be relevant to every app store review):<br>a) **Population** of the group of apps being examined; what do the apps have in common?<br>b) What type of **interventions** / activities are being used by the app? c) What types of **comparison** apps are being analyzed (if appropriate)? d) What are the stated **outcomes** of the apps being analyzed?<br>e) What type of systematic, theoretical framework is being used by the app?	A comparator is not relevant to the present study. We have used the TECH approach to determine eligibility criteria for the systematic review of commercially available health apps. Reference: Conducting a systematic review and evaluation of commercially available mobile applications (apps) on a health-related

	topic: the TECH approach and a step-by-step methodological guide. Doi: 10.1136/bmjopen-2023-073283. PMID: 37308269; PMCID: PMC10277147.
**METHODS**<br><br>5. Indicate if a review protocol has been published or is otherwise publicly accessible, if and where it can be accessed (e.g., Web address), and, if available, provide registration information including registration number.	The protocol is registered in the INPLASY. INPLASY2024100035, DOI 10.37766/inplasy2024.10.0035<br>Protocol is published in JMIR Research Protocol Journal with doi: 10.2196/71071
6. Specify app characteristics; refer to adapted PICOS model for app store reviews again if appropriate; and report other characteristics (e.g., app availability, app cost, app user ratings, app language, app development information) used as criteria for eligibility, giving rationale.	Yes
7. Describe all information sources (e.g., app store types with dates of coverage, contact with app developers to identify research [particularly published research in peer-reviewed forums] and confirmation of developers' associations and credentials) in the search, and dates of the search, including start and end dates.	Yes
8. Present full electronic search strategy for at least one app store, including any limits used (as far as the app store search options will allow), such that it could be repeated. Note any differences in the search options available between different app stores.	Yes
9. State the process for selecting apps (i.e., screening, eligibility, included in systematic review, and, if	Yes. The apps were downloaded and used in

applicable, included in the meta-analysis). This should state whether apps were reviewed based on their store descriptions, or if apps were individually downloaded and verified through actual use.	real-time to review rather than just focusing on the app store description.
10. Describe method of data extraction from apps (e.g., more than one investigator duplicating the process) and any processes for obtaining and confirming data from investigators.	Yes
11. List and define all variables for which data were sought (e.g., PICOS, funding sources) and any assumptions and simplifications made.	Yes
12. Describe methods used for assessing risk of bias of individual apps (e.g., has the research on efficacy and effectiveness been completed by authors with an association to the app), and how this information is to be used in any data synthesis or critical analysis.	One of the 350 reviewed apps is developed and tested for usability by the corresponding author. Since none of the names of the apps are mentioned in the manuscript and individual app- level data/names are not reported, the authors have not discussed regarding the risk of bias of the above-mentioned app. During the final review of the manuscript, the authors will disclose the information of the above-mentioned single app.
13. State the principle summary measures and descriptive statistics, including the limits of such statistics.	Yes

14. Describe the methods of handling data and combining results of studies, if relevant. If conducting a meta-analysis, include an assessment of consistency.	NA. This protocol includes a review of mental health apps themselves. It does not include a review of studies on apps.
15. Specify any assessment of risk of bias that may affect the cumulative research evidence that is located after conducting the app store review (e.g., publication bias, selective reporting within studies, independence / non-independence of research authors etc.).	NA (as mentioned in item no. 14)
16. Describe methods or aspects of additional analyses that may be unique to the present app store review, if appropriate, indicating why they are appropriate and stating their purpose.	Yes
**RESULTS**	
17. Give numbers of apps screened, assessed for eligibility, and included in the review, with reasons for exclusions at each stage, ideally with a flow diagram.	Yes (Refer to Figure. 1)
18. For each app, present characteristics for which data were extracted (e.g., targeted purpose of each app, developer characteristics, theoretical framework of the app, PICOS etc.). Provide a list, in a Table within the main body of the text if the list is small, or in an Appendix or accessible online format if the list is large.	Yes (Refer to Table. 3) Note: The table highlights characteristics of all the apps in terms of frequency and percentages.
19. Present data on risk of bias of each app and, if available, any outcome level assessment (see item 12).	One of the 350 reviewed apps is developed and tested for usability by the corresponding author. Since none of the names of the apps are mentioned in the

	manuscript and individual app- level data/names are not reported, the authors have not discussed regarding the risk of bias of the above-mentioned app. During the final review of the manuscript, the authors will disclose the information of the above-mentioned single app.
20. In identifying literature for evidence claimed by an app, provide summary data for intervention groups proportionate to the aims of the app store review (e.g., demographic statistics, effect estimates and confidence intervals, etc.). If appropriate, consider using visual summary techniques, such as forest plots.	NA
21. If relevant, present results of each meta-analysis done, including confidence intervals and measures of consistency.	NA
22. Present results of any assessment of risk of bias across apps, and/or across any research that has been found relating to the final list of apps in the review (see Item 15).	Yes
23. If appropriate, give results of additional analyses (see Item 16).	Yes
**DISCUSSION**	
24. Summarize the main findings including the strength of evidence for each app. Consider the relevance of findings in relation to the industry / sector that this group of apps belongs, being as precise and focused as possible. If appropriate, rather than stating the implications of findings in a general sense (e.g., for the "health industry"), narrow the focus (e.g., for the "treatment of diabetes").	Yes
25. Discuss limitations at study and outcome level (e.g., risk of bias), and at review level (e.g., limitations of search options in app stores, reporting bias). Authors of the review should provide a disclosure of their role in any	Yes

developmental capacity or association with any apps within the group of apps being reviewed.<br><br>26. Provide a general interpretation of the results in the context of other evidence, and implications for future research.<br><br>FUNDING<br><br>27. Describe sources of funding for the app store review and other support (e.g., supply of data); role of funders for the systematic review (e.g., did the funders specify a preferred publication route).	 <br>Yes<br><br>Yes
**Section 2: Evaluating an App Store Systematic Review (adapted from AMSTAR 2) (This manuscript is a systematic review of mental health apps in app stores; the authors have indicated their responses based on the parameters to report findings)**<br><br>1. Did the research questions and inclusion criteria for the app store review include the components of (adapted PICOS model for app store reviews): a) **Population** of the group of apps being examined; what do the apps have in common?<br>b) What type of **interventions** / activities are being used by the app? c) What types of **comparison** apps are being analyzed (if appropriate)? d) What are the stated **outcomes** of the apps being analyzed?<br>e) What type of **systematic**, theoretical framework is being used by the app?<br><br>2. Did the report of the app store review contain an explicit statement that the review methods were established prior to the conduct of the review (for example, by having its protocol published), and did the report justify any significant deviations from the protocol?	A comparator is not relevant to the present study.<br>We have used the TECH approach to determine eligibility criteria for the systematic review of commercially available health apps.<br>(doi: 10.1136/bmjopen-2023-073283. PMID: 37308269; PMCID: PMC10277147.)<br><br>Yes

3. Did the app store review authors explain their selection of the app designs for inclusion in the review?	Yes
4. Did the app store review authors use a comprehensive search strategy for their app store search?	Yes
5. Did the app store review use multiple reviewers to select relevant apps, and use an adequate consensus strategy in shortlisting apps?	Yes
6. Was data extraction adequate for meeting the app store review's aims, and did more than one reviewer duplicate the data extraction?	Yes
7. Did the app store review authors provide a list of excluded apps, or make a statement that such a list was available upon request? Were exclusions justified?	Yes
8. Did the app store review authors describe the included apps in adequate detail?	Yes
9. Did the app store review authors define the parameters of their search, including dates of their search, which app stores were searched, and in which country they performed their search?	Yes
10. Did the app store review authors report on the sources of funding for their review studies?	Yes
11. Did the app store review authors use appropriate methods for statistical analysis of results in line with the aims of their study?	Yes
12. Did the app store review authors adequately report on or make a statement about the sources of each health app's development (if relevant to the aims of the study).	Yes
13. Did the app store review authors list the key words that were used for each search so that the same search can be duplicated by readers of the study, as well as providing a rationale for using these keywords?	Yes
14. Did the app store review include the names of the shortlisted apps (in the case where a small number of	No

apps were shortlisted), or a statement from the authors agreeing to provide this information upon request (in the case where many apps were shortlisted whose names could not be included due to limitations of journal space)?	
15. Did the app store review authors make a statement about claims of published research evidence by shortlisted apps? Did the authors confirm the existence of such claimed research by doing a literature search?	Yes
16. Did the app store review authors report any potential sources of conflict of interest, including if they are involved in the development of health apps, or if they stand to gain financially or otherwise from the proceeds or success of a health app?	Yes
